# Microspheres targeted with a mesothelin antibody and loaded with doxorubicin reduce tumor volume of human mesotheliomas in xenografts

**DOI:** 10.1186/1471-2407-13-400

**Published:** 2013-09-11

**Authors:** Sherrill L Macura, Jeremy L Steinbacher, Maximilian B MacPherson, Melissa J Lathrop, Mutlay Sayan, Jedd M Hillegass, Stacie L Beuschel, Timothy N Perkins, Page C Spiess, Albert van der Vliet, Kelly J Butnor, Arti Shukla, Marilyn Wadsworth, Christopher C Landry, Brooke T Mossman

**Affiliations:** 1 Department of Pathology, University of Vermont College of Medicine, 89 Beaumont Avenue, Burlington, VT 05405-0068, USA; 2 Department of Chemistry, University of Vermont, 82 University Place, Burlington, VT 05405-1706, USA; 3 Departments of Medicine, University of Vermont College of Medicine, 89 Beaumont Avenue, Burlington, VT 05405-0068, USA

**Keywords:** Targeted therapy, Mesoporous silica, Peritoneum, Chemotherapy, Microparticles

## Abstract

**Background:**

Malignant mesotheliomas (MMs) are chemoresistant tumors related to exposure to asbestos fibers. The long latency period of MM (30-40 yrs) and heterogeneity of tumor presentation make MM difficult to diagnose and treat at early stages. Currently approved second-line treatments following surgical resection of MMs include a combination of cisplatin or carboplatin (delivered systemically) and pemetrexed, a folate inhibitor, with or without subsequent radiation. The systemic toxicities of these treatments emphasize the need for more effective, localized treatment regimens.

**Methods:**

Acid-prepared mesoporous silica (APMS) microparticles were loaded with doxorubicin (DOX) and modified externally with a mesothelin (MB) specific antibody before repeated intraperitoneal (IP) injections into a mouse xenograft model of human peritoneal MM. The health/weight of mice, tumor volume/weight, tumor necrosis and cell proliferation were evaluated in tumor-bearing mice receiving saline, DOX high (0.2 mg/kg), DOX low (0.05 mg/kg), APMS-MB, or APMS-MB-DOX (0.05 mg/kg) in saline.

**Results:**

Targeted therapy (APMS-MB-DOX at 0.05 mg/kg) was more effective than DOX low (0.05 mg/kg) and less toxic than treatment with DOX high (0.2 mg/kg). It also resulted in the reduction of tumor volume without loss of animal health and weight, and significantly decreased tumor cell proliferation. High pressure liquid chromatography (HPLC) of tumor tissue confirmed that APMS-MB-DOX particles delivered DOX to target tissue.

**Conclusions:**

Data suggest that targeted therapy results in greater chemotherapeutic efficacy with fewer adverse side effects than administration of DOX alone. Targeted microparticles are an attractive option for localized drug delivery.

## Background

Malignant mesothelioma (MM) is an aggressive tumor of mesothelial cell origin and is often associated with occupational exposures to asbestos fibers [1-3]. MM predominantly develops in the pleural and peritoneal cavities, and tumors often spread diffusely throughout these cavities before a diagnosis is made in late stages of tumor development. Current therapies for MM include resection by surgery, systemic chemotherapy, gene therapy, immunotherapy, radiation, and palliative procedures [4]. The limited response and deleterious effects of current treatment therapies demand the development of novel therapeutic strategies to decrease the systemic toxicity of chemotherapeutic agents and specifically target MMs. Intraperitoneally (IP) delivered chemotherapeutics following tumor resection is an actively explored approach to decrease systemic toxicity and enhance the uptake of chemotherapeutic drugs [5,6]. It has recently been reported that patients who were administered hyperthermic IP chemotherapy (DOX in combination with cisplatin) following surgical debulking of peritoneal MMs had an increased 5 year survival rate of 29-63% [7]. Peritoneal MMs are of unique importance because of their increasing prevalence in younger adults often without obvious exposure to asbestos [8].

Effective treatment of MM remains an unmet clinical need. We have previously characterized an IP model of MM in severe combined immunodeficient (SCID) mice [9], and have evaluated the potential of acid-prepared mesoporous silica particles (APMS) for their ability to target and be retained by spheroid and mesenteric MMs *in vivo* following modification with an antibody specific to human mesothelin (APMS-MB) [10]. APMS microparticles (patented by Christopher C. Landry at the University of Vermont) are amorphous silica particles (1-2 μm diameter) with a disordered pore structure, a large specific surface area, and a large pore volume [11]. Characteristics such as tunable particle diameter and pore size, the large internal surface area, and the ability to functionalize the external surfaces of APMS with tetraethylene glycol (TEG) or antibodies to facilitate targeting and uptake of the particles by cells, make APMS an optimal delivery agent for chemotherapeutic agents, DNA plasmids, siRNA, or other macromolecules [12-14]. Additionally, amorphous silicas produce no chronic adverse biological responses [15]. Recently we have shown that APMS-injected IP penetrate to the interior of MMs over time without changes in immune profiles in peritoneal lavage fluid (PLF) [10].

In this study, we targeted particles to MM using an antibody for mesothelin, a 40 kD glycophosphatidylinositol-anchored glycoprotein on the cell surface that normally functions in cell-to-cell adhesion [16]. Mesothelin is a differentiation antigen with expression normally limited to mesothelial cells lining the pleura, pericardium, and peritoneum [16,17]. However, mesothelin is over-expressed in several human cancers including virtually all MMs, ovarian cancers (70% of cases), lung cancers (50% of cases), and pancreatic/biliary adenocarcinomas [18-22]. The 71 kD protein encoded by the mesothelin gene is further processed to a 31 kD protein, megakaryocyte potentiating factor, which is released into serum [18,19,23]. The expression of mesothelin in the serum of MM patients results in the production of mesothelin-specific immunoglobulin G (IgG) antibodies, enabling a protective, host humoral immune response [20].

After IP injection, APMS functionalized with an antibody specific to the membrane-bound mesothelin protein (APMS-MB) are more readily taken up, internalized, and retained by MMs over time when compared to non-antibody functionalized APMS [10]. Particle uptake by major organs is low compared to tumor uptake when examined by inductively coupled plasma mass spectrometry (ICP-MS) or scanning electron microscopy and energy dispersive spectroscopy. Moreover, we have characterized urinary clearance patterns using gadolinium-labeled APMS in healthy rats [24] as well as selective and active uptake of APMS functionalized with a number of moieties, including TEG, fluorophores, and targeting antibodies in mesothelial and mesothelioma cells *in vitro*[12-14]. Based on results of studies demonstrating that APMS do not elicit toxicity nor immune responses after intrapleural or IP injections [10,14], we hypothesized that targeted APMS-MB loaded internally with DOX (APMS-MB-DOX) would inhibit MM development and growth more effectively than DOX alone. In studies here, we demonstrate the efficacy of this approach and examine the effects of this strategy on tumor volume and weight, animal weight and health, tumor cell necrosis and proliferation, and changes in inflammatory cell profiles in PLF. Our findings are not only relevant to MM, but also to treatment of other intracavitary tumors (ovarian, pancreatic) that over-express mesothelin.

## Methods

### Cells and cell culture

The HMESO MM line was previously described [25] and obtained from Joseph R. Testa (Fox Chase Cancer Center, Philadelphia PA). HMESO cells were maintained in Dulbecco’s Modified Eagle Medium DMEM/F12 50/50 (Mediatech, Manassas, VA) supplemented with 10% fetal bovine serum (FBS), 0.1 μg/mL hydrocortisone (Sigma, St. Louis, MO), 2.5 μg/mL insulin, 2.5 μg/mL transferrin, 2.5 ng/mL sodium selenite (Sigma, St. Louis, MO) and penicillin-streptomycin (50 U/mL penicillin G, 50 μg/mL streptomycin sulfate) (Invitrogen, Carlsbad, CA) [26]. Cells were maintained at 37°C in 5% CO_2_.

### Synthesis of acid-prepared mesoporous spheres (APMS) and subsequent modifications

Porous, amorphous silica microparticles (APMS) pre-modified with TEG on their external surfaces [11], were used for subsequent modifications in experiments described here [13]. APMS microparticles were modified using an antibody to mesothelin (MB) and loaded with DOX as previously described [13,24].

### SCID mouse xenograft model of human malignant mesothelioma

**Figure 1:**
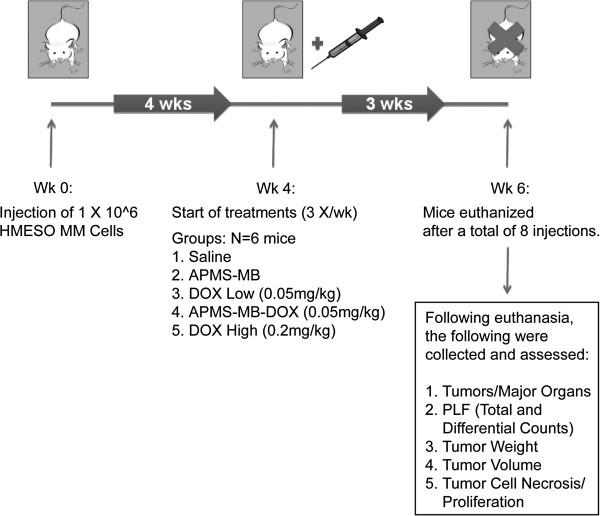
**Model of MM tumor induction and targeted chemotherapy in SCID mice.** A schematic overview of the MM tumor induction protocol. Tumor formation was achieved by a single IP injection of MM (1 × 10^6^ cells) followed by a 4 wk latency period to allow tumor growth. Mice were weighed every 3-4 days. The treatment groups included IP injections of saline (500 μL), APMS-MB (no DOX), DOX at a low dose (0.05 mg/kg), APMS-MB loaded with DOX at equivalent low dose (0.05 mg/kg), and DOX at a high dose (0.2 mg/kg) (all in saline at 500 μL, 3× per wk for 3 wks). Mice were euthanized after a total of 8 treatment injections due to the morbidity and weight loss in the DOX high group (N = 6 mice/group). Two mice in the saline group did not develop tumors, thus N = 4 in subsequent analysis of data.

The SCID mouse xenograft model has been previously described [9,26]. Figure 1 shows the treatment regimen employed here. Groups consisted of mice receiving saline (0.9% NaCl {pH 7.4}), APMS-MB (no DOX), DOX high (0.2 mg/kg), DOX low (0.05 mg/kg), or APMS-MB-DOX (0.05 mg/kg) (n = 6 mice/treatment group). In brief, 1 × 10^6^ HMESO cells (in 50 μL sterile 0.9% NaCl {pH 7.4}) were injected IP into each mouse. All mice were weighed 3×/wk for 4 wks prior to treatments. Both free-floating spheroid and mesenteric tumors lining the diaphragm develop at 4 wks in all mice [9], at which time IP treatments were initiated. Mice were weighed on each treatment day prior to injection to enable the adjustment of dose per individual body weight. All animal procedures were approved by the University of Vermont IACUC committee.

### Measurement of tumor weight and volume

Mesenteric and spheroid tumors from treated mice were weighed and measured using digital calipers following euthanasia and resection. Free-floating spheroid tumors were weighed individually if possible, but in cases where spheroids were too small, all the spheroids (< 1 mm^3^) for an individual animal were pooled and measured as one mass. Tumor volumes were calculated following the formula (l × w × h × pi/6). The average tumor volume and weight per mouse was then calculated for each treatment group. We also evaluated the percentage distribution of tumors at different sites (Table 1). Gross examination of tumors and major organs was performed following euthanasia.

**Table 1:** **Percent (%) MM tumor distribution**^**a**^

	**Saline**	**APMS-MB**	**DOX low (0.05 mg/kg)**	**APMS-MB-DOX (0.05 mg/kg)**	**DOX high (0.2 mg/kg)**
Injection site	4.17	2.38	4.17	5.56	0.00
Pleura	0.00	0.00	3.33	0.00	0.00
Diaphragm	4.76	0.00	0.00	0.00	0.00
Esophagus	0.00	0.00	0.00	2.78	2.38
Liver	10.91	5.16	2.78	4.86	11.62
Kidney	4.44	3.97	6.11	9.33	8.49
Gut	8.33	19.60	12.36	15.56	4.17
Stomach	11.67	13.10	18.06	15.63	7.45
Spleen	4.52	14.21	11.25	20.38	7.04
Intestine	26.43	28.89	25.00	14.64	36.47
Bladder	0.00	0.00	0.00	0.00	2.78
Ovaries/Testes/Vas deferens	24.76	12.70	16.94	11.27	19.60

^a^As measured by the average percent (%), per treatment group, of the number tumors recovered found attached to or adjacent to respective organs and areas.

### Histopathology

Mesenteric and spheroid tumors collected from saline controls and animals treated with DOX low (0.05 mg/kg) or APMS-MB-DOX (0.05 mg/kg) were preserved in 4% paraformaldehyde, paraffin embedded, and processed for hematoxylin and eosin (H&E) staining. Processing and sectioning (4 μm) of tissues was performed in the Department of Pathology (Fletcher Allen Health Care, Burlington, VT). Staining of tissues using H&E was performed following standard protocols [27], and MM tissue sections were examined for necrosis by a board-certified pathologist (KJB). Images were captured using an Olympus BX50 upright light microscope (Olympus America, Lake Success, NY) with an attached Q Imaging Retiga 2000R digital CCD camera (Advanced Imaging Concepts, Inc., Princeton, NJ).

### Ki-67 immunohistochemistry and quantification

Mesenteric and spheroid tumor sections were de-paraffinized in xylenes (2 × 15 min) and rehydrated to ddH_2_O through a graded series of ethanol (100% to 50%). Staining of tissues for Ki-67 as a marker of proliferation was performed following standard protocols as previously described [28]. The monoclonal mouse anti-Human Ki-67 primary antibody (Vector Laboratories, Burlingame, CA, and Leica Microsystems, Buffalo Grove, IL) (1:25 in 1% bovine serum albumin (BSA) in 1× phosphate buffered saline (PBS)) was used [29]. Slides were then dehydrated by dipping them in 100% ethanol (5×) twice, xylenes (5×), and then allowed to air dry before they were coverslipped using Permaslip® (American MasterTech, Lodi, CA) as the mounting agent. Cell proliferation, measured as the percentage of Ki-67 positive cells was quantitated by counting the number of Ki-67 positive (brown nuclei) and negative (purple nuclei) tumor cells, in 5 random Ki-67 expressing regions consisting of 200 cells each. Representative images were captured using an Olympus BX50 upright light microscope (Olympus America, Lake Success, NY) with an attached Q Imaging Retiga 2000R digital CCD camera (Advanced Imaging Concepts, Inc., Princeton, NJ).

### Staining for M1 and M2 tumor-associated macrophages (TAMs)

To determine patterns of populations of TAMs associated with tumor masses, slides cut from frozen OCT blocks of saline and APMS-MB groups were fixed in acetone for 15 min and stained as described previously (10), but using the primary antibodies, rabbit anti-mouse NOS II (1:1000 dilution) for M1 TAMs and rat anti-mouse CD206 (1:200 dilution) for M2 TAMs. Both antibodies were from AbD, Serotec (Raleigh, NC). The secondary antibodies used were goat anti-rabbit Alexa 555 and goat anti-rat Alexa 488. Both secondary antibodies were diluted 1:500 and purchased from Invitrogen (Carlsbad, CA). Nuclei were stained with 4,′6-diamidino-2-phenylindole (DAPI) (1:200 dilution). All antibody and DAPI dilutions were in PBS/1% BSA. Slides were coverslipped as described previously (10) and examined with a confocal laser scanning microscope (CLSM) Zeiss 510 META (Thornwood, NY). Tiled images (5 images × 5 images) were used to assess profiles of TAMs in whole MMs, and representative areas were examined at higher magnifications (10).

### Collection of PLF samples from SCID mice and preparation of cytospins

Mice were euthanized with 0.1 mL of Sleep Away (26% sodium pentobarbital, Webster Veterinary) before 5 mL of cold phosphate buffered saline (PBS) (Ca/Mg-free) was instilled into the peritoneal cavity of each mouse using an 18 gauge needle. The abdomen was then lightly massaged, and the PBS removed. PLF was centrifuged, and cell free supernatant was stored at -80°C. Total white blood cell counts in PLF were assessed manually using a hemocytometer. Cytospins were prepared from 50,000 cells following standard protocols [14,26] and were stained using a HEMA 3 kit (Fisher Scientific, Middletown, VA) per the manufacturer instructions. Three hundred cells per slide were counted by two individuals. The counts were then averaged, and the percentages of each cell type and total numbers were used to calculate the total numbers of cell type/mL for each animal in each treatment group.

### Determination of DOX concentrations in PLF using high pressure liquid chromatography (HPLC)

After collection of PLF fluid as previously described [9], PLF samples (500 μL) from the saline, DOX low (0.05 mg/kg) and APMS-MB-DOX (0.05 mg/kg) treatment groups were centrifuged at 10,000 × g at 4°C for 5 min. The supernatant was transferred to a fresh Eppendorf vial, and a daunorubicin internal control (final concentration 1 μM) was added to samples. Samples were then vortexed briefly and incubated at 37°C for 15 min in the dark. Proteins were precipitated from PLF samples by adding 250 μL of acetone and 50 μL of aqueous ZnSO_4_ solution (400 mg/mL) followed by vortexing. Samples were then centrifuged at 20,000 × g (10 min) at 4°C, and the supernatants were transferred to new vials, and dried under a fume hood in a heat block (65°C) under a stream of nitrogen. The residue was solubilized in 1 mL of methanol, and 200 μL of sample was used for HPLC analysis.

### Determination of DOX concentration in tumor tissue using HPLC analysis

A section of tumor tissue from one tumor/animal from the saline, DOX low (0.05 mg/kg) and APMS-MB-DOX (0.05 mg/kg) treatment groups was removed, and the wet weight recorded. The tissue was then placed in a vial of 380 μL PBS, and 10 μL of lysis solution (Triton X-100, 3% final concentration) was added. Tissue was homogenized using a Biospec Tissue-Tearor (Biospec Products, Racine, WI). Ten μL of Proteinase K (10 mg/mL) was added to the homogenized tissue followed by incubation for 45 min in a water bath at 65°C. The internal standard, daunorubicin, was added to each sample (112.8 μL of 10 μg/mL solution), and samples were brought to a final volume of 800 μL with 1 × PBS. Ten μL of phenylmethylsulfonyl fluoride was added to samples for 10 min. Twenty μL MgCl_2_ (0.4 M) and 40 μL DNase I (1 mg/mL) were then added, and samples were centrifuged (10,000 × g) for 5 min at 4°C followed by incubation in a water bath at 37°C for 30 min. Following incubation, 450 μL of each sample was added to 450 μL methanol and 45 μL ZnSO_4_ (400 mg/mL), vortexed, and centrifuged (10,000 × g) for 5 min at 4°C, whereupon 200 μL was used for HPLC analysis.

### HPLC

The HPLC method for detection of DOX described previously [9,30] was followed for the analysis of all samples.

### Statistical analysis

The General Linear Models procedure (PROC GLM) of the SAS System for Windows was used for statistical analysis of data. All data (excluding the analysis of weights over time) were evaluated primarily by one way analysis of variance (ANOVA) followed by Fisher’s LSD pairwise tests for adjustment of multiple pair-wise comparisons between treatment groups. For the weights across time data, a repeated measures analysis of variance was performed on mouse weights to compare the rate of weight loss among treatment groups. Statistical significance was determined as p ≤ 0.05.

## Results

### Tumor volume is significantly decreased after treatment with APMS-MB-DOX (0.05 mg/kg) in contrast to DOX (0.05 mg/kg) alone or saline controls

**Figure 2:**
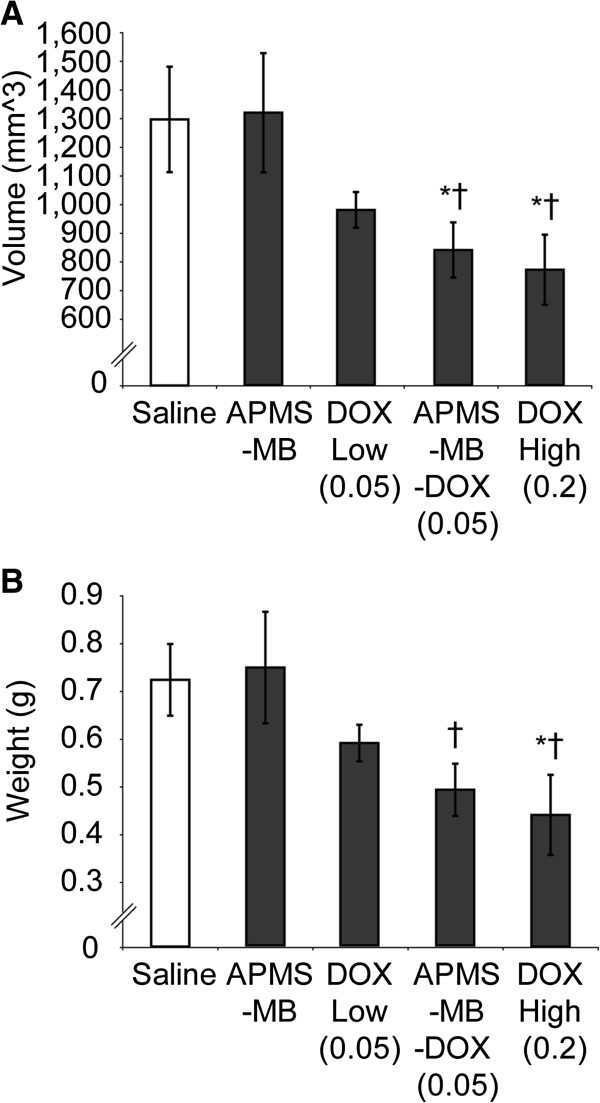
**Treatment with APMS-MB-DOX microparticles (0.05 mg/kg) significantly reduces tumor volume and weight. (A)** The average tumor volume (mm^3^), including pooled spheroids, per treatment group is provided (Mean +/- SEM). Treatment with DOX at high concentrations (0.2 mg/kg) or APMS-MB-DOX (0.05 mg/kg) significantly reduced the average tumor volume/mouse compared to saline (*) and APMS-MB (†) controls (p ≤ 0.05). **(B)** The average tumor weight (gm) is represented for each treatment group (Mean +/- SEM). Treatment with DOX high (0.2 mg/kg) significantly reduced the average tumor weight/mouse compared to saline (*) and APMS-MB (†) controls (p ≤ 0.05). Treatment with APMS-MB-DOX (0.05 mg/kg) significantly reduced tumor weight in comparison to the APMS-MB group (†).

We hypothesized that targeted treatment using APMS-MB-DOX would reduce MM tumor volume and weight more effectively than the same dose of DOX administered alone in an established IP xenograft model [9,26] (Figure 1). After 3 wks of treatment, we observed that mice receiving IP injections of DOX at 0.2 mg/kg (high concentration) had significantly reduced tumor volumes (p ≤ 0.05) compared to saline treated mice, those receiving DOX at 0.05 mg/kg (low concentration), or those receiving APMS-MB without DOX (Figure 2A). Mice receiving APMS-MB-DOX at 0.05 mg/kg also showed reduced tumor volume compared to the same groups (p ≤ 0.05). Consistent with these results, average tumor weight was significantly reduced (p ≤ 0.05) in the DOX high (0.2 mg/kg) and the APMS-MB-DOX low (0.05 mg/kg) groups when compared to saline controls. Treatment with DOX (0.05 mg/kg) alone, or APMS-MB groups did not have an effect on tumor weight in comparison to saline controls (Figure 2B). These results demonstrate that treatment with APMS-MB-DOX (0.05 mg/kg) significantly reduces tumor weight in contrast to the DOX (0.05 mg/kg) group alone. Moreover, they suggest that changes in tumor weight and volume are due to more efficient delivery of DOX and not due to the particles (APMS-MB) themselves. In addition, we evaluated the distribution of MMs and their metastases to distal organs (Table 1). These studies revealed that the vast majority of tumor metastases occurred in the intestines or ovaries/testes (approximately 25% of MMs at each site) in untreated (saline control) mice. These numbers were reduced most dramatically (15 and 11%, respectively) in the APMS-MB-DOX (0.05 mg/kg) group.

### Weight loss associated with high DOX therapy (0.2 mg/kg) is not observed at effective APMS-MB-DOX (0.05 mg/kg) concentrations

**Figure 3:**
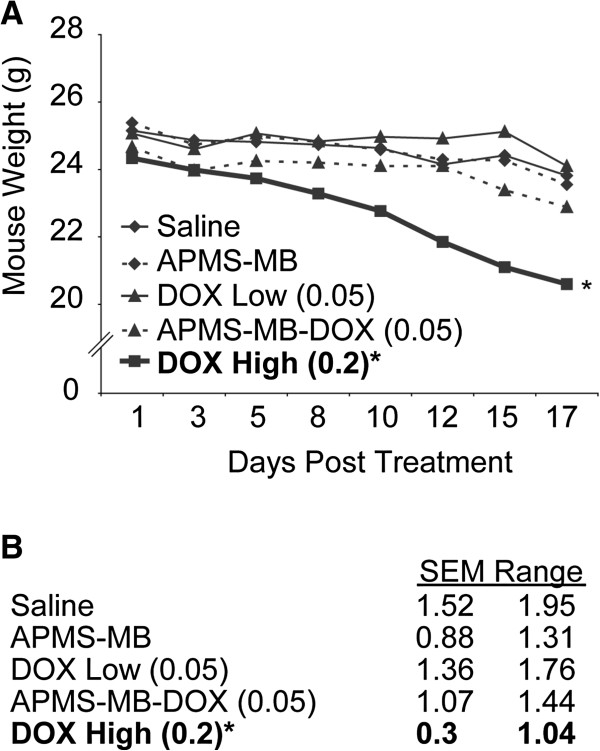
**Treatment with DOX high (0.2 mg/kg) causes toxicity and weight loss.** Mice were weighed prior to each treatment. **(A)** Weights are plotted as the mean group weight (gram) at each treatment time point. Differences in the slope of average weights over time in the DOX high (0.2 mg/kg) treatment group were significantly lower (*) (p ≤ 0.05) in comparison to all other treatment groups. No significant differences in weight were found between all other treatment groups. **(B)** SEM ranges for each treatment group over time are provided.

Once tumors become large (> 2.0 cm^3^), MM-bearing mice rapidly lose weight, become lethargic, and cease normal grooming behavior. We hypothesized that modification of the APMS microparticles with a targeting MB would increase drug delivery to tumor tissues and decrease DOX-related weight loss. The weights of mice used in this study were measured throughout the experiment. Indeed, mice treated at the lower, but effective dose of APMS-MB-DOX (0.05 mg/kg) had less average weight loss than those treated with the high dose (0.2 mg/kg) of DOX alone (Figure 3). Pair-wise comparisons of weight loss rates also showed that the treatment group receiving the higher dose of DOX (0.2 mg/kg) had a significantly different slope (*) than other treatment groups. Thus, mice receiving a high dose of DOX alone lost significantly more weight over time in contrast to mice receiving a lower non-effective dose of DOX alone (0.05 mg/kg), or mice receiving APMS-MB-DOX (0.05 mg/kg). These results suggest that an effective dose of APMS-MB-DOX is well tolerated over the duration of multiple treatments.

### Treatment with APMS-MB-DOX (0.05 mg/kg) is associated with trends toward increased necrosis and significantly decreased proliferation in spheroid MMs

**Figure 4:**
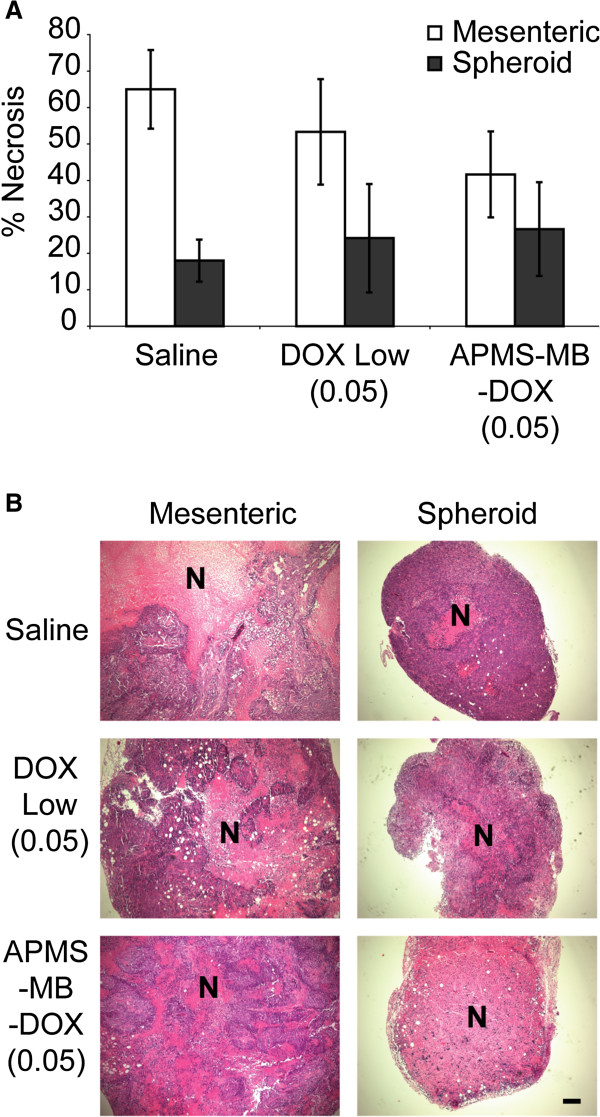
**Administration of APMS-MB-DOX microparticles (0.05 mg/kg) enhances cell death in spheroid tumors.** Paraffin sections (4 μm thick) of mesenteric and spheroid MM tumors from each animal were stained using H&E. **(A)** Quantification of the average percent necrosis (Mean +/- SEM) per area of section was assessed by a board-certified pathologist analyzing 4 fields at 10× magnification. Trends in mesenteric tumors did not reach statistical significance. **(B)** Representative images of both mesenteric and spheroid tumors; scale bar represents 100 μM. N = necrotic area.

**Figure 5:**
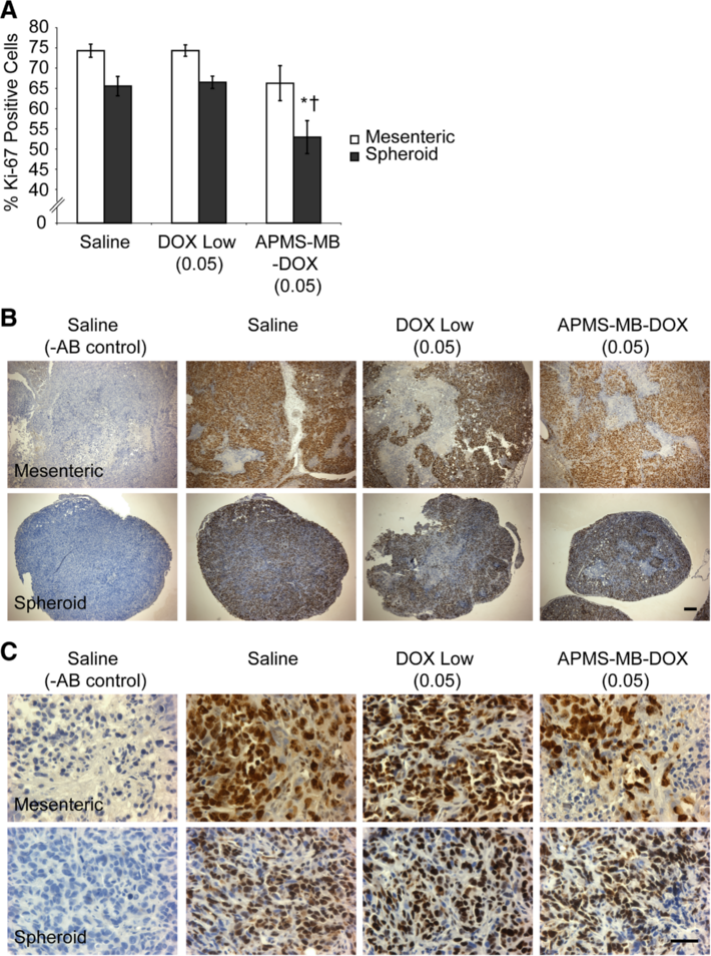
**Administration of APMS-MB-DOX microparticles (0.05 mg/kg) decreases proliferation in spheroid tumors. (A)** Sections were stained for Ki-67 to assess cell proliferation. Quantification of Ki-67-positive cells per total cells counted from the same tumors (Mean +/- SEM) was scored by two individuals evaluating 5 regions at 40× magnification and counting 200 cells/region for each tumor section using a blind coding system. Treatment with APMS-MB-DOX (0.05 mg/kg) significantly decreased Ki-67-positive cells in spheroid MMs compared to saline controls (*) or groups receiving a comparable dose of DOX alone (†) (p ≤ 0.05). **(B)** Representative images of tissues stained for Ki-67 (4× objective); scale bar represents 100 μM. **(C)** Higher magnification micrographs were acquired with a 40× objective. Scale bar represents 50 μM.

Gross examination of the tumors and major organs in the peritoneal cavity following euthanasia revealed that mice in all groups had MM involvement with the intestine, reproductive organs, stomach, gut, kidney, liver and spleen (Table 1). Animals treated with DOX high (0.02 mg/kg) or DOX low (0.05 mg/kg) also had bloody PLF. Bloody PLF was not observed in mice treated with APMS-MB-DOX (0.05 mg/kg). Given the reduction in tumor volume, decreased weight loss, and the lack of bloody PLF in groups treated with APMS-MB-DOX (0.05 mg/kg) in comparison to the same dose of DOX alone, APMS-MB or saline controls, we hypothesized that tumor tissue from these animals would have increased areas of necrosis and fewer proliferating cells. We stained representative mesenteric and spheroid (free-floating) tumors [9] from each animal using H&E staining in the saline, DOX low (0.05 mg/kg) and APMS-MB-DOX (0.05 mg/kg) treatment groups, and a board-certified pathologist assessed the tissues to evaluate the average percentage of total tumor area exhibiting necrosis. Examination of mesenteric tumors revealed more necrotic tissue in the saline treatment group - an average of 65% compared to the DOX (0.05 mg/kg) alone (53%) or APMS-MB-DOX (41%) groups. However, these trends were not statistically significant. In spheroid tumors, necrosis was roughly comparable between treatment groups (20-25%) and lower overall (Figure 4A, B). No significant changes in the percentage of Ki-67-positive, i.e., proliferating cells, were observed in mesenteric MMs (Figure 5A-C). However, Ki-67-positive cell staining in spheroid MMs showed significantly decreased proliferation in the APMS-MB-DOX (0.05 mg/kg) treatment group when compared to saline controls or mice receiving a comparable dose of DOX alone (0.05 mg/kg) (p ≤ 0.05). Histopathology of spheroids showed focal areas of necrosis in the interiors of these smaller, ascites-like tumors. Thus APMS-MB-DOX may have a greater impact on inhibition of proliferation because microparticles are in the IP milieu and penetrate and access the tumor interior more easily [10].

### Treatment with APMS-MB-DOX (0.05 mg/kg) causes significant changes in inflammatory cell profiles in PLF

**Figure 6:**
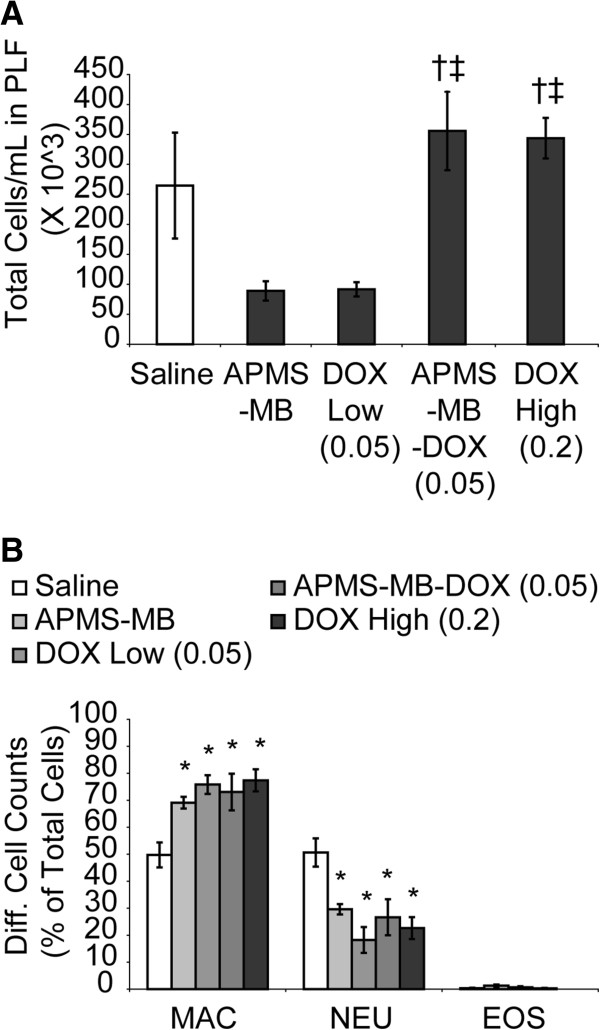
**Effects of treatment on total and differential cell populations in PLF. (A)** Total cells recovered in PLF samples (Mean +/- SEM) were increased in the APMS-MB-DOX (0.05 mg/kg) and DOX high (0.2 mg/kg) groups as compared to APMS-MB (†) and DOX low (0.05 mg/kg) groups (‡) (p ≤ 0.05). **(B)** All treatments significantly increased the percentage of total macrophage (MAC) cells/mL (Mean +/- SEM) and significantly decreased the percentage of total neutrophils (NEU/mL) compared to saline control groups (*) (p ≤ 0.05).

We have speculated, based on many findings, that chronic inflammation is a feature of the development and establishment of MMs [31]. To address this question, and to determine whether or not the treatment regimens used in these experiments affected immune cell profiles in PLF, cytospins were evaluated as described previously [9]. PLF samples from treatment groups receiving effective concentrations of APMS-MB-DOX (0.05 mg/kg) or DOX alone (0.2 mg/kg) contained a significantly higher (p ≤ 0.05) total number of cells/mL in PLF than mice treated with DOX (0.05 mg/kg) alone or APMS-MB (Figure 6A). Differential cell counts revealed that when compared to the saline group, the percentage of macrophages was significantly higher in all treatment groups, whereas numbers of neutrophils were significantly lower (p ≤ 0.05) (Figure 6B).

### HPLC analysis confirms delivery of DOX to tumor tissue

To confirm that the reduction in tumor volume and weight was due to DOX, we examined levels of DOX in tumor homogenates in tissues and PLF fluids of tumor-bearing mice receiving 3× weekly doses of DOX (0.05 mg/kg) or APMS-MB-DOX (0.05 mg/kg). HPLC analysis was performed at the end of 3 wks of treatment (data not shown). We were unable to detect DOX in the PLF of either treatment group, suggesting it had been released and already excreted and/or accumulated in tumor tissue. Supporting the latter hypothesis, DOX was detected in the tumor tissues of treatment groups receiving DOX alone (2.47 nmoles/gm of MM tissue) or APMS-MB-DOX (2.57 nmoles/gm of MM tissue). Though the amount of DOX was slightly higher in the MMs in the targeted therapy group, the amounts of DOX were not significantly different between groups.

Historically in patients receiving DOX for chemotherapy, cardiotoxicity has been a documented side-effect. We purposely designed APMS in the micrometer rather than nano- particle size range so that they would not enter the vasculature and deliver DOX to the heart. Moreover, *in vivo* imaging of Gadolinium-labeled APMS microparticles in rodents after IP injection using MRI shows that particles not remaining in the IP space are cleared via the bladder. In these studies and others, examination of the hearts of mice by a board-certified pathology failed to show any particles or adverse pathology.

### Both M1 and M2 tumor-associated macrophages (TAMs) occur in MMs

**Figure 7:**
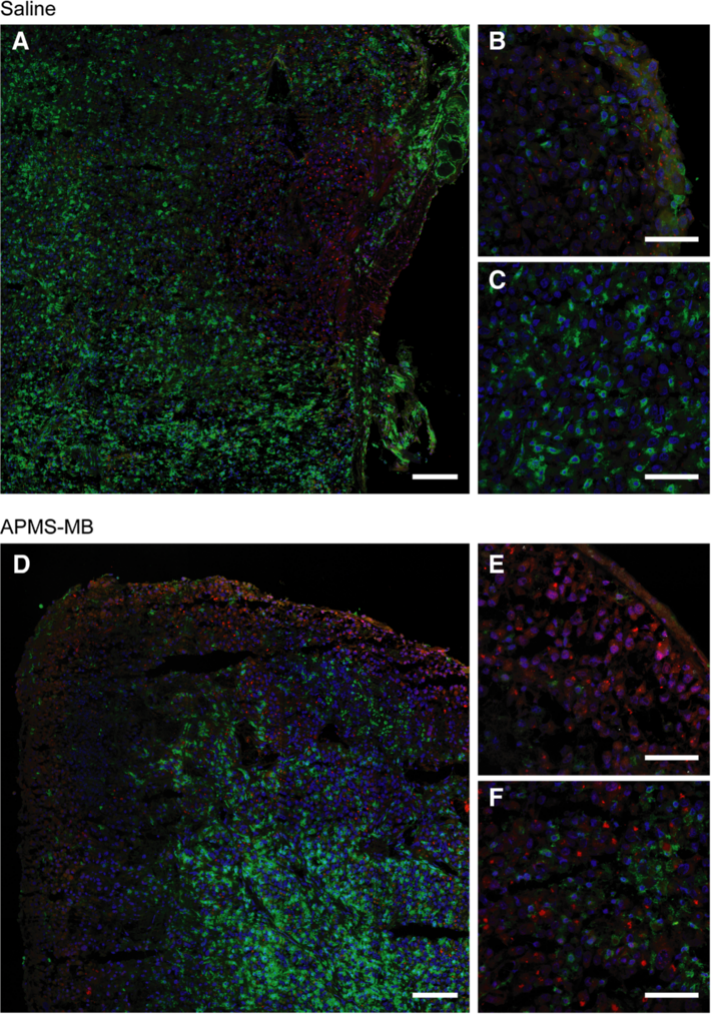
**Localization of M1 and M2 tumor-associated macrophages (TAMs) in tumors. (A)** Tiled images from saline (untreated) MM. **(B)** Higher magnification of the surface of a saline (untreated) MM. **(C)** Interior of a saline (untreated) MM. **(D)** Tiled image of a MM treated with APMS-MB. **(E)** Higher magnification of the surface of APMS-MB-treated MM. **(F)** Interior of an APMS-MB-treated MM. Note that nuclei are stained blue, M1 TAMs are red, and M2 TAMs are green. White scale bars in **A** and **D** are 100 μM and in **B**, **C**, **E** and **F** are 50 μM.

MMs are historically associated with areas of inflammation in both animal models and human tissues, and TAMs also are a prominent feature of our SCID mouse model [10]. In studies here, our objective was to determine if TAMs in MMs reflected M1 (anti-tumor) and/or M2 (pro-tumor) phenotypes. In both untreated (saline) and APMS-MB mice, M2 (green) TAMs appeared to predominate (Figure 7), but M1 TAMs (red) were noted in discrete surface accumulations along the edges of tumors. M2 were found more frequently in the interior of tumors. Further quantitative studies are planned in all treatment groups.

## Discussion

Clinical trials that have been conducted or are ongoing have utilized anti-mesothelin recombinant immunotoxins (SS1 {dsFv} PE38: Fv portion of antibody SS1 fused to truncated *Pseudomonas* exotoxin) alone and in combination with chemotherapeutics [32-34], or the anti-mesothelin antibody MORAb-009 (a chimeric IgG/k/SS1 {dsFv} fusion antibody) leading to an antibody-dependent cell-mediated cytotoxic response [18,35]. Additionally, tumor vaccines targeted against mesothelin are currently being studied. A mesothelin tumor vaccine (CRS-207) is currently in a phase II trial in combination with the pancreatic cancer vaccine GVAX [36], and a pre-clinical *in vitro* study has reported the success of a lentivirus-expressing anti-mesothelin microRNA (MSLNmiR3) that silences the mesothelin gene (MSLN). This approach causes a 60% reduction in ovarian cancer cell (OVca429) viability following infection with (MSLNmiR3) [37].

We show in Figure 2 that APMS-MB-DOX (0.05 mg/kg) significantly reduces tumor volume compared to untreated (saline) tumors, whereas free DOX at the same concentration did not achieve significance in reduction of tumor volume. We also have previously evaluated in triplicate experiments in this same tumor model, groups of SCID mice exposed to APMS alone (160 mg/kg), free DOX alone at 3 concentrations (0.33, 1 and 5 mg/kg) and APMS-DOX (no mesothelin-labeling) at 0.33 and 1 mg/kg doses by Hillegass et al. [26]). Comparison of these results shows that APMS-MB DOX (0.05 mg/kg) when compared to APMS- DOX (0.33 mg/kg) is effective therapeutically, even at > 6-fold lower concentrations. Furthermore, APMS-MB-DOX (0.05 mg/kg) significantly reduced tumor volume and weight when compared to mice treated with only APMS-MB. We have shown here that with the targeting abilities of APMS-MB-DOX, we can achieve similar decreases in tumor volume and weight reduction by using only 1/4 of the DOX dose (0.05 mg/kg) when compared to saline controls. These findings demonstrate that enhanced delivery of DOX via APMS-MB microparticles is causing enhanced efficacy rather than an immune response triggered by the presence of the MB [20]. This conclusion is further supported by the specific detection of DOX in tumor tissues by HPLC. These encouraging results at low doses of DOX highlight the need for future experiments that could examine the efficacy of multiple doses of APMS-MB-DOX. APMS-MB may also be used to deliver other chemotherapeutic drugs such as Alimta and the combination of cisplatin/carboplatin, the gold standard in chemotherapeutic treatment of MM.

In addition to the decreases in tumor volume and weight observed using APMS-MB-DOX microparticles, the animals in this group lost less body weight than mice treated with effective doses of high DOX (0.2 mg/kg) alone. Mice receiving APMS-MB-DOX were also less lethargic and maintained normal activity and grooming behavior when compared to mice treated with DOX alone at either high or low doses. Thus studies here suggest that local administration of lower doses of therapeutic drugs or macromolecules [12] that can be encapsulated in APMS is an effective method to circumvent toxicities associated with systemic administration of agents [26]. Local administration of hyperthermic chemoperfusion [6,7,38-41] is a treatment regimen for patients with diffuse peritoneal or pleural MM, thus it is feasible to perfuse drug-loaded APMS into the pleural or peritoneal cavities.

Histochemical analysis of tumor tissue from the saline, low DOX (0.05 mg/kg) alone and APMS-MB-DOX (0.05 mg/kg) treatment groups was performed on both mesenteric and spheroid tumors to evaluate the mechanisms of tumor shrinkage in the latter group. Treatment with APMS-MB-DOX (0.05 mg/kg) caused significant decreases in cell proliferation, as evaluated by Ki-67 staining, in spheroid tumors. It was also noted that in both mesenteric and spheroid tumors treated with targeted APMS-MB-DOX, areas of necrosis had a more diffuse cellular pattern compared to the very discrete and more centralized areas of necrosis seen in other treatment groups (see Figure 4B). This appears to reflect infiltration of cells of the immune system, such as tumor associated macrophages (TAMs) of the M1 (anti-tumor) or M2 (pro-tumor) phenotype, which may modulate tumor cell death. We have previously noted large numbers of TAMs in both spheroid and mesenteric MMs [10], and these data are supported by photomicrographs of M1 and M2 macrophages in MMs and increased macrophages observed in PLF fluids as reported here.

TAMs have been shown to play a very important role in tumor cell invasion, proliferation, survival, angiogenesis, and immune suppression [42,43]. While macrophages from normal tissue are capable of presenting tumor antigens, lysing tumor cells, activating the anti-tumor functions as well as activating T cells and natural killer cells, TAMs in solid tumors may lack these functions. Moreover, in a hypoxic tumor microenvironment, TAMs can undergo further modification of function and or modulation/suppression of immunostimulatory cytokines [44]. The decreases in neutrophils seen in PLF in all treatment groups suggest that increased numbers of macrophages localized to tumors causes TAMs to assume an immunosuppressive phenotype and suppress the infiltration of neutrophils [45].

We also have observed that mesenteric tumors recovered from saline-treated mice tend to have larger areas of necrosis at their centers, and we hypothesize that this may be due to the hypoxic microenvironment existing deeper in the tissue that is typical of solid tumors [46,47]. This may explain why trends towards necrosis are greater in untreated animals as these tumors are generally larger (data not shown). The most striking effect of APMS-MB-DOX is observed in smaller spheroids where the extracellular matrix and tumor vasculature are most likely developing at the time of treatment [48]. Since solid tumors are a dynamic system, the composition and architecture of the tissue, the binding of drugs to cellular components, and changes in tumor cell density over time may all play a role in the effect of a given therapy on developing tumors [49]. Spheroid MMs treated with APMS-MB-DOX contained significantly lower percentages of proliferating cells compared to either saline-treated controls or groups after treatment with DOX alone at the same dose. This may reflect the fact that APMS are able to penetrate tumor tissue and traverse to the interior of the tumor mass over time. In support of this concept, APMS-MB persist both extracellularly and intracellularly in tumor cells and macrophages in PLF [10].

In our IP model of MM, neutropenia and increases in macrophages in PLF correlate with tumor growth over time [9]. Using this model, we have also characterized time-dependent patterns of inflammation and tumor development using multiple MM cell lines. Several known growth factors for MMs including Vascular Endothelial Growth Factor (VEGF), Granulocyte Colony Stimulating Factor (G-CSF), basic Fibroblast Growth Factor (bFGF), and Platelet Derived Growth Factor (PDGF-BB) are significantly elevated (p ≤ 0.05) in PLF before development of MMs [9]. It is possible that APMS-MB-DOX via direct interactions between MB and these proteins inhibits their expression or activity, thus decreasing cell proliferation. Thus, MB alone might be potentially inactivating gene or protein expression of chemokines or cytokines, avenues which are presently being explored.

## Conclusions

In conclusion, we demonstrate here that use of targeted microparticles (APMS-MB-DOX) (0.05 mg/kg) is an effective therapeutic strategy that is superior to treatment with equal amounts of DOX (0.05 mg/kg) alone and decreases the detrimental side-effects of systemic administration of DOX (0.2 mg/kg) alone. Using this targeting approach, effective doses of DOX can be reduced approximately 4-fold without any side effects or toxicity. Since APMS-MB-DOX (0.05 mg/kg) has the potential to decrease the number of proliferating tumor cells in developing MM spheroids, this approach also may be useful when pre-malignant or malignant MM cells are first seen in peritoneal or pleural fluids, i.e., before histopathological diagnosis of MMs.

## Abbreviations

APMS: Acid-prepared mesoporous silica particles; ANOVA: Analysis of variance; MSLNmiR3: Anti-mesothelin microRNA; M1: Anti-tumor associated macrophages; bFGF: Basic Fibroblast Growth Factor; BSA: Bovine serum albumin; DOX: Doxorubicin; FBS: Fetal bovine serum; PROC GLM: General Linear Models procedure; G-CSF: Granulocyte Colony Stimulating Factor; H&E: Hematoxylin and eosin; HPLC: High pressure liquid chromatography; IgG: Immunoglobulin G; ICP-MS: Inductively coupled plasma mass spectrometry; IP: Intraperitoneal; MM: Malignant mesothelioma; MSLN: Mesothelin gene; MB: Mesothelin-specific antibody; PLF: Peritoneal lavage fluid; PBS: Phosphate buffered saline; PDGF-BB: Platelet Derived Growth Factor; M2: Pro-tumor associated macrophages; SCID: Severe combined immunodeficient mice; TEG: Tetraethylene glycol; TAMs: Tumor associated macrophages; VEGF: Vascular Endothelial Growth Factor.

## Competing interests

CCL patented APMS through UVM. The other authors declare they have no competing interests.

## Authors’ contributions

SLM performed or supervised all experiments and drafted this paper. SLM and BTM designed all experiments. JLS, MJL, JMH, AS and CCL provided input in design and interpretation of results. MBM, MS, SLB, TNP and MJL assisted in animal experiments. JLS and CCL synthesized various microparticles. PCS and AvdV performed HPLC analyses. KJB assisted in histopathology and assessment of tumor necrosis. All authors read and approved the final manuscript.

## Authors’ information

BTM is the Director of the Environmental Pathology Training Program at UVM, a recipient of the Wagner Award presented by the International Mesothelioma Interest Group for scientific contributions to research on MM, and an awardee of the Career Achievement Recognition Award for Scientific Accomplishments from the American Thoracic Society.

## Pre-publication history

The pre-publication history for this paper can be accessed here:

http://www.biomedcentral.com/1471-2407/13/400/prepub
